# Splenectomy for hypersplenism with or without preoperative splenic artery embolisation

**DOI:** 10.1186/s41747-018-0053-6

**Published:** 2018-09-12

**Authors:** Mohamed M. A. Zaitoun, Mohammad Abd Alkhalik Basha, Ahmed Raafat, Tamer Rushdy, Walid A. Mawla

**Affiliations:** 1 Diagnostic Radiology Department, Zagazig University, Zagazig, Egypt; 2 General Surgery Department, Zagazig University, Zagazig, Egypt

**Keywords:** Embolisation, therapeutic, Hypersplenism, Splenectomy, Splenic artery, Splenomegaly

## Abstract

**Background:**

Although splenectomy is considered the preferred treatment for hypersplenism, intraoperative blood loss remains a common occurrence. We prospectively compared the perioperative and clinical outcome of splenic artery embolisation (SAE) before open splenectomy (OS) versus OS alone in two concurrent patient groups.

**Methods:**

From January 2016 to January 2018, 50 patients with hypersplenism underwent combined SAE and OS (study group). For comparison, we considered 50 age- and gender-matched case controls undergoing OS without prior SAE during the same period (control group). Perioperative and clinical outcomes were compared between the two groups. Mann–Whitney *U* test, Student’s t-test, χ^2^ or Fisher’s exact test were used as appropriate.

**Results:**

No significant differences were found between the two groups for age, gender and laboratory investigations (*p* ≥ 0.250). Mortality rate was zero in both groups. No patients of the study group needed perioperative blood transfusion in comparison with patients of the control group (*p* = 0.003). A significant increase in platelet count was noted in the study group after SAE compared to the control group (*p* = 0.024). No significant differences between the two groups were observed for operating time, postoperative complications and postoperative stay (*p* ≥ 0.237).

**Conclusion:**

We confirm that preoperative SAE in patients who undergo splenectomy for hypersplenism significantly reduces the need for blood transfusion in comparison to splenectomy without prior embolisation. Preoperative SAE is a safe procedure with neither morbidity nor mortality.

## Key points

- Splenic artery embolisation before splenectomy reduced the complications of the procedure
- Splenic artery embolisation before splenectomy decreased the blood loss during the operation
- When splenic artery embolisation is performed before splenectomy, there is no need for blood transfusion

## Background

Hypersplenism occurs due to hyperactivity of the spleen and defined as a triad of splenomegaly, pancytopenia and normocellularity of bone marrow [1–3]. Surgical splenectomy is a traditional treatment for hypersplenism; however, splenectomy is associated with significant postoperative complications [4]. Splenic artery embolisation (SAE) has been widely used as an alternative to splenectomy for the treatment of hypersplenism because it is minimally invasive and associated with fewer complications [5–8].

Preoperative SAE makes splenectomy a safer procedure by reducing the intraoperative blood loss and the need for blood transfusion [9]. SAE before splenectomy improves the platelet count, reduces the size of the spleen and makes it softer [10, 11].

Several reports in the literature have investigated the role of SAE before splenectomy. We reported herein a combined treatment approach to splenomegaly due to hypersplenism consisting of preoperative SAE performed by an interventional radiologist and open splenectomy (OS) performed by a general surgeon with 6–8 h between the two procedures. The purpose of this study was to evaluate the clinical outcome of this combined treatment approach in the management of hypersplenism and compared with OS alone.

## Methods

### Study design

We performed a prospective observational study in two concurrent groups of patients receiving or not receiving SAE before OS.

### Ethical considerations

The present study was approved by the institutional review board. The risk and potential benefits of the procedures were explained to all patients and a written informed consent was obtained. The study was performed in accordance with the ethical principles of the Declaration of Helsinki.

### Study population

This study was conducted on 50 consecutive patients admitted to the Surgical Department of the Zagazig University Hospital Zagazig, Egypt, from January 2016 to January 2018 with clinically and laboratory proven hypersplenism (mean platelet count < 40,000 u/μL). All patients of the study group underwent a combined treatment including SAE before OS. For the purpose of study comparison, we selected another 50 patients out of 197 patients (25.4%) admitted to the same Surgical Department during the same period who underwent OS without prior SAE (control group). The patients of the control group were selected to be age- and gender-matched to those in the study group (for each patient of the study group, we selected the first one with the same gender and with an age included in an interval of ± 2.5 years compared with that of the patient of the study group). The patients in the control group underwent OS alone without prior SAE based on the surgeon’s decision, the choice of patients or they were individuals with known allergy to iodined contrast agent. Patients with hypocellular or infiltrative bone marrow disease, ischemic heart disease, renal failure or malignant disease and those with advanced liver disease were excluded from the study.

### Patient assessment

On admission, all patients were subjected to a detailed history, thorough clinical examination, laboratory and biochemical investigations (including bone marrow aspiration), abdominal ultrasonography and color-coded duplex scanning of the portal circulation. For each patient, splenic volume was measured twice using ultrasound, before SAE and just before OS. Ultrasound measurement of the spleen volume was performed according to the following protocol: ultrasound was performed using an S40 Exp/S40 Pro/S40/S35 Digital Color Doppler US system with a 3.5/5-MHz convex transducer probe. Spleen metrics were assessed by using defined standard algorithms [12]. With the patient in the supine position, the examination started in the posterior axillary line in the approximate area of the tenth rib through an intercostal space to identify the longitudinal view of the spleen with the hilum. In this position, maximum length and width were measured. Respiratory manoeuvres sometimes helped improve the visibility of the spleen. Turning the probe over the hilum by 90° from the plane of maximal spleen length provided the transverse image to measure the spleen anteroposterior dimension [13]. The haematological assessment, including platelet count, was done preoperatively just before the OS and on day 10 after surgery. A strict protocol of prophylactic parenteral therapy was followed, with a ten-day course of intravenous injection of 1000 mg cefazoline at an interval of 6 h (three days before SAE and seven days after OS). All patients received pneumococcal vaccination before the procedure. Patient demographics are listed in Table 1.

**Table 1:** Demographics and co-morbidities of patient groups

		Study group (*n* = 50)	Control group (*n* = 50)	*p* value
Age (years, mean ± SD (range))		38 ± 8.1 (28–62)	34 ± 7.8 (25–60)	0.893
Gender (n (%))	Male	23 (46)	20 (40)	0.686
	Female	27 (54)	30 (60)	
Splenic size (cm, mean ± SD (range))		16.2 ± 3.9 (13–26)	16.4 ± 4.8 (13–25)	0.685
Child-Pugh class (n (%))	A	47 (94)	48 (96)	
	B	3 (6)	2 (4)	
	C	0	0	
Co-morbidities (n (%))	DM	7 (14)	6 (12)	0.659
	Cholestasis	17 (34)	15 (30)	0.365
	Hypertension	3 (6)	4 (8)	0.786
CBC (mean ± SD (range))				
Platelet (U/μL)		8500 ± 2444 (6500–18,600)	9200 ± 2765 (6500–17,400)	0.646
WBCs (U/μL)		2700 ± 549 (1150–3200)	2580 ± 642 (1190–3140)	0.413
HB (gm/dL)		8.3 ± 0.79 (7.5–10.8)	8.5 ± 0.82 (7.8–10.4)	0.250
Liver function tests (mean ± SD (range))				
ALT (U/μL)		72.8 ± 3.5 (67.2–79.5)	73.4 ± 3.7 (65.8–78.6)	0.359
AST (U/μL)		70.6 ± 2.1 (66.8–79.2)	71.2 ± 2.9 (67.4–77.9)	0.329
TB (μmol/L)		23.4 ± 3.3 (18.5–29.4)	22.8 ± 3.2 (18.9–28.6)	0.257

*OS* open splenectomy, *SAE* splenic artery embolisation, *DM* diabetes mellitus, *CBC* complete blood count, *WBC* white blood cell, *HB* haemoglobin, *ALT* alanine aminotransferase, *AST* aspartate aminotransferase, *TB* total bilirubin

### Splenic artery embolisation

**Fig. 1:**
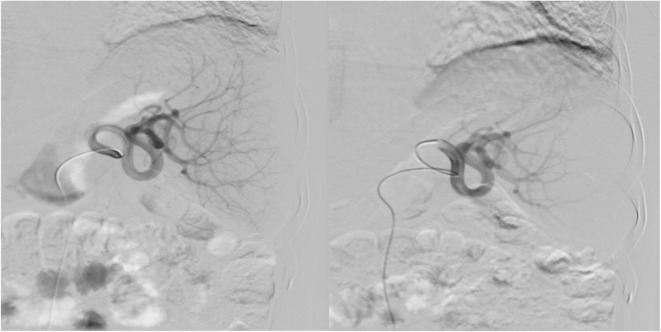
Digital subtraction angiography of the splenic artery before (*left panel*) and after (*right panel*) embolisation, showing 60–70% occlusion of the parenchymal vascularity of the spleen

The SAE procedure was performed 6–8 h before OS by an interventional radiologist with ten years of experience in SAE using the following technique: the femoral artery was punctured and a 5-F introducer sheath was placed; the splenic artery was catheterised under fluoroscopy using a 4- or 5-F cobra catheter (Imager, Boston Scientific, USA). We used a microcatheter (Renegade HI-FLO microcatheter, Boston Scientific, USA) in five patients with a tortuous splenic artery. The microcatheter was advanced through the cobra catheter till the splenic hilum. After securing the left gastroepiploic artery to preserve distal pancreatic branches of the splenic artery, infusion of polyvinyl alcohol particles (300–355 μm in diameter) (Contour Emboli, Boston Scientific Cork Ltd., Ireland) mixed with 25 mL of iodinated contrast and one ampule of gentamycin (80 mg) was done. The embolisation procedure was considered satisfactory when at least 60–70% of the parenchymal vascularity appeared as occluded at subjective assessment (Fig. 1). The procedure was performed using an angiography unit (Cath Lab System, Siemens, Artis Zee Ceiling VC21C). The average duration of the procedure was 20 min.

### Open splenectomy

The OS was performed by three surgeons with more than ten years of experience. An upper midline incision was done. Upon entry into the abdominal cavity, dissection was performed with the blunt and sharp technique. The surgeon’s hand followed the convex surface of the organ and identified the peritoneal attachments. The spleen was gently grasped and displaced medially toward the incision. The avascular peritoneal attachments and ligaments were incised, but in patients with portal hypertension, any ligaments were ligated. At the hilum, the splenic artery and veins were identified, carefully dissected, doubly ligated with 0 silk, and transfixed by 2–0 silk suture ligatures. To avoid injury to the pancreas, the dissection was carried out at the hilum in close proximity to the spleen. Next, the short gastric vessels were identified and ligated. After removal of the spleen, haemostasis was obtained and confirmed in a systematic fashion through careful inspection of the left sub-phrenic area, the greater curvature of the stomach and the short gastric vessel area, as well as the splenic hilum. Inspection of these areas was facilitated by proper retraction of the stomach and small bowel to allow clear visualisation of the left upper quadrant and surgical beds. Attention was then turned to the surgical field to check for active bleeding. When active bleeding was identified, we achieved haemostasis. Drains were not routinely used, except in cases where an injury of the tail of the pancreas was suspected or confirmed. The abdominal incision was closed by approximating the linea alba using 1–0 polypropylene monofilament sutures in a continuous fashion. The skin was closed with staples.

### Statistical analysis

All statistical analyses were performed using SPSS v16.0 (SPSS, Inc., Chicago, IL, USA). Baseline clinical and laboratory data that might determine different postoperative outcomes were compared between the two groups. Categorical variables were presented as frequencies. Continuous variables were expressed as mean ± standard deviation or median and interquartile range (IQR) according to their normal or non-normal distribution, respectively. Mann–Whitney *U* test, Student’s t-test, χ^2^ or Fisher’s exact test were used as appropriate. A *p* value < 0.05 was considered statistically significant.

## Results

We did not find significant differences between the two groups for age, gender and laboratory investigations (see Table 1).

Perioperative details are given in Table 2. After a 6–8 h interval between SAE and OS, 72% of patients in our study showed reduced splenic volume. A significant difference was observed between the two groups concerning the perioperative increase in the platelet count. A significant difference was detected between the two groups concerning the perioperative need for blood transfusion; there was no need for blood transfusion in the study group while 45/50 (90%) of controls received blood transfusion.

**Table 2:** Perioperative outcomes

	Study group (*n* = 50)	Control group (B) (*n* = 50)	*p* value
Operating time (min, mean ± SD)	151 ± 23	174 ± 35	0.564
Platelet count between SAE and OS (U/μL mean ± SD (range))	70,000 ± 28,255 (13000–190,000)	9200 ± 2765 (6500–17,400)	0.024
Perioperative need for transfusion (%)	0/50	45/50 (90)	0.003
Splenic size (cm, mean ± SD (range))	16.2 ± 3 (12–25)	16.4 ± 4.8 (13–25)	<0.001
Change of spleen volume after SAE (n (%))			
Decrease	36 (72)		
No changes	14 (28)		
Increase	0		

*OS* open splenectomy, *SAE* splenic artery embolisation

Minor postoperative complications occurred in the two groups (Table 3). Portal vein thrombosis was identified in two patients of the study group, one month after surgery.

**Table 3:** Postoperative outcomes

		Study group (*n* = 50)	Control group (*n* = 50)	*p* value
Postoperative stay (days, mean ± SD)		9.5 ± 3.2	9.6 ± 4.1	0.237
Platelet count on day10 (U/μL, mean ± SD (range))		380,000 ± 12,359 (160,000–540,000)	340,000 ± 13,927 (120,000–490,000)	0.255
Complications (%)	Pleural effusion	0/50 (0)	1/50 (2)	
	Pulmonary infection	1/50 (2)	3/50 (6)	
	Postoperative bleeding	0/50 (0)	2/50 (4)	
	Portal vein thrombosis	2/50 (4)	0/50 (0)	
	Deep venous thrombosis	1/50 (2)	0/50 (0)	
	Incision infection	0/50 (0)	0/50 (0)	
	Total	4/50 (8)	6/50 (12)	0.739
Mortality rate		0/50	0/50	1.000

In addition, portal vein thrombosis was identified in two patients of the study group, one month after surgery. *SD* standard deviation

The mortality rate was zero in both groups.

## Discussion

Surgical splenectomy has been performed since 1950 for hypersplenism caused by portal hypertension. Portal hypertension, complicated by gastrointestinal haemorrhage, has become one of the principal indications for splenectomy [14, 15]. However, the risk of intraoperative blood loss and haemorrhagic complications associated with splenectomy in patients with portal hypertension has been recognised in many series [16, 17]. Poulin et al. [18] and Fujitani et al. [19] suggested the possibility of reducing the incidence of intraoperative bleeding by performing preoperative SAE.

The value of preoperative SAE observed in our study for a better control of intraoperative blood loss during splenectomy confirms the results of previously published studies in the management of patients with hypersplenism [10, 11], as there was no need for blood transfusion in the SAE patient group. In 2007, Bau et al. [10] reported on a patient series without preoperative SAE. They had severe haemorrhagic complications that required transfusions of platelets in 65% of cases and of red blood cells in 47%, while there was no need for blood transfusion in their preoperative SAE group. In 2012, Wu et al. [11] reported that only 10% of the SAE patient group needed blood transfusion, compared to 30% in the group of laparoscopic splenectomy without preoperative SAE and 52% in the group who underwent OS splenectomy without preoperative SAE.

Of note, Iwase et al. [20] mentioned a significant difference in favour of SAE patient group concerning the perioperative increase in the platelet count, attributing this to the reduction of splenic function. In our study, there was an overall improvement in the platelet count after embolisation in the SAE patient group (mean 380,000 U/ μL, on day 10 post surgery). This finding provides a better evidence on the effect of the SAE and confirms other many older series [5, 10, 11, 21]. SAE leads to a softer and smaller spleen, improves the surgical view and reduces the incidence of bleeding [11, 22].

There is no consensus in the literature about the optimal time interval between the SAE and the splenectomy. The time interval was in the range of 2 h to one day in the published studies. Wu et al. [11] showed that it is effective to increase the interval between embolisation and surgery to reduce splenic volume. An interval of 2.8–4.8 h between SAE and laparoscopic splenectomy was sufficient to reduce the splenic volume in 80% of patients in their series. In our study, 72% of patients in our study showed reduced splenic volume after a 6–8 h interval between SAE and OS.

In our opinion, perioperative SAE makes splenectomy safer. Even though many studies of pre- or perioperative SAE showed good results, the technique has yet to become widespread. This may be partly due to the fact that complications secondary to the injection of microparticles have been described [11, 23, 24]. Regarding the two cases of portal vein thrombosis, they did not complain of any discomfort. Low-molecular-weight heparin was used to prevent thrombopoiesis and a regular colour Doppler ultrasound was performed three months after OS.

Our study has an important limitation. Cases were selected for preoperative SAE or direct splenectomy depending on the surgeon’s opinion and/or the patient’s preference rather than being based on objective guidelines or being randomised. This may have led to treatment bias. However, the characteristics of the two groups were quite similar as shown in Table 1.

In conclusion, we confirm that preoperative SAE in patients who undergo splenectomy for hypersplenism significantly reduces blood transfusion in comparison to splenectomy without prior embolisation.

## Abbreviations

OS: Open splenectomy
SAE: Splenic artery embolisation
